# Room-Temperature Self-Healable Blends of Waterborne Polyurethanes with 2-Hydroxyethyl Methacrylate-Based Polymers

**DOI:** 10.3390/ijms24032575

**Published:** 2023-01-29

**Authors:** Ioanna Tzoumani, Zacharoula Iatridi, Athena M. Fidelli, Poppy Krassa, Joannis K. Kallitsis, Georgios Bokias

**Affiliations:** 1 Department of Chemistry, University of Patras, GR-26504 Patras, Greece; 2 Megara Resins Anastassios Fanis S.A., Vathi Avlidas, GR-34100 Evia, Greece

**Keywords:** waterborne polyurethane, 2-hydroxyethyl methacrylate, *N*,*N*-dimethylacrylamide, glycidyl methacrylate, contact angle, water uptake, self-healing

## Abstract

The design of self-healing agents is a topic of important scientific interest for the development of high-performance materials for coating applications. Herein, two series of copolymers of 2-hydroxyethyl methacrylate (HEMA) with either the hydrophilic *N*,*N*-dimethylacrylamide (DMAM) or the epoxy group-bearing hydrophobic glycidyl methacrylate were synthesized and studied as potential self-healing agents of waterborne polyurethanes (WPU). The molar percentage of DMAM or GMA units in the P(HEMA-co-DMAMy) and P(HEMA-co-GMAy) copolymers varies from 0% up to 80%. WPU/polymer composites with a 10% *w*/*w* or 20% *w*/*w* copolymer content were prepared with the facile method of solution mixing. Thanks to the presence of P(HEMA-co-DMAMy) copolymers, WPU/P(HEMA-co-DMAMy) composite films exhibited surface hydrophilicity (water contact angle studies), and tendency for water uptake (water sorption kinetics studies). In contrast, the surfaces of the WPU/P(HEMA-co-GMAy) composites were less hydrophilic compared with the WPU/P(HEMA-co-DMAMy) ones. The room-temperature, water-mediated self-healing ability of these composites was investigated through addition of water drops on the damaged area. Both copolymer series exhibited healing abilities, with the hydrophilic P(HEMA-co-DMAMy) copolymers being more promising. This green healing procedure, in combination with the simple film fabrication process and simple healing triggering, makes these materials attractive for practical applications.

## 1. Introduction

Currently, the demand for sustainable and long-lasting materials that can self-repair after physical damage, and thus, extend their service life, leads to the development of an emerging class of smart materials, namely self-healing materials [1,2,3,4]. Self-healing mechanisms can be categorized in two types: the extrinsic self-healing which is based on pre-embedded healing agents [5,6], and the intrinsic self-healing mechanism in which the materials are capable of repairing mechanical damage through reversible covalent or non-covalent bonding between molecular chains. Some examples of reversible covalent bonds [7] are Diels-Alder reactions [8,9], imine bonds [10], disulfide bonds [11,12,13], and boron ester bonds [14,15], while examples of reversible non-covalent bonds comprise hydrogen bonds [16,17,18], host-guest interactions [19], metal-ligand coordination [20], and ionic interactions [21]. Concerning non-covalent mechanisms, hydrogen bonding interactions are considered among the most promising candidates for self-healing and find numerous applications in biomedical fields, or in the industry of coatings and adhesives [22,23]. Several articles in literature report the incorporation of hydrogen-bonding units in polymeric materials or coatings toward enhancing self-healing ability [24,25,26,27].

Poly(2-hydroxyethyl methacrylate) (PHEMA) is an inexpensive, biocompatible, water-insoluble, and non-degradable polymer bearing hydroxyl functional groups. The abovementioned characteristics combined with other advantages like stimuli-responsiveness, chemical stability, transparency, or elastomeric properties, make PHEMA homopolymers and PHEMA-based materials excellent candidates for use in biomedical and industrial applications [28] including fabrication of soft contact lenses and controlled release of drugs [29,30], wound healing [31], bone tissue regeneration [32], biosensors [33,34], immobilization of proteins and enzymes [35], separation of molecules [36], metal-ion adsorption [37], or as self-healing agents [38,39,40,41].

Poly(*N*,*N*-dimethylacrylamide) (PDMAM) is a nonionic, hydrophilic polymer with interesting properties. Due to its water solubility and biocompatibility, it can be used as a co-monomer for several applications such as superabsorbent materials [42], biomedical [43], or water remediation applications [44,45]. Poly(2-hydroxyethyl methacrylate-co-*N*,*N*-dimethylacrylamide) (P(HEMA-co-DMAM)) copolymers are smart materials that combine the characteristics of both their HEMA and DMAM counterparts, and can be used in numerous applications as heavy metals and dyes adsorbents [46,47], sensors and biosensors [48,49], ingredients in interpenetrating polymer network (IPN) hydrogels with improved mechanical properties [50].

Glycidyl methacrylate (GMA) is a low-cost and non-toxic monomer bearing highly reactive epoxy groups that can be coupled with groups like carboxylic acids, amines or thiols, via ring opening reactions [51]. Many studies report the incorporation of epoxy groups through the copolymerization of HEMA with the monomer GMA. Poly(2-hydroxyethyl methacrylate-co-glycidyl methacrylate) (P(HEMA-co-GMA)) copolymers have been used in biotechnology as protein, enzyme, or other molecules binders/immobilizers [52,53,54,55,56], in applications concerning heavy metal ions and dye adsorption [57,58,59]. To our knowledge, there are no articles reporting examples of P(HEMA-co-GMA) copolymers with self-healing properties.

In a previous work, we reported the use of GMA-based copolymers as potential healing agents of waterborne polyurethane (WPU) films [60]. The incorporation in these WPUs of copolymers of GMA with either n-butyl acrylate (BA) or poly(ethylene glycol) methyl ether methacrylate (PEGMA) led to homogenous composite films. For the healing study, heating at 80 °C or addition of water on the scratched films were applied as potential external healing triggers. It was shown that these copolymers are effective materials for the water-triggered healing of WPUs films. Herein, in an attempt to design copolymers with additional and potentially improved healing abilities, we present a series of HEMA-based copolymers with either the hydrophilic monomer DMAM or the epoxy group-bearing hydrophobic monomer GMA. Depending on the copolymer series, these copolymers are expected to combine characteristics like improved hydrophilicity, dynamic hydrogen bonding due to the hydroxyl (-OH) functionality of HEMA and amide groups of DMAM, and post modification reactions of the epoxy rings of GMA. WPU/polymer films, were prepared after mixing, casting, and thermally treating the copolymers with WPU resins. The properties of the films, including water contact angle measurements and water uptake, were evaluated and corelated with the water-mediated healing abilities observed.

## 2. Results and Discussion

### 2.1. Copolymers Characterization

**Figure 1:**
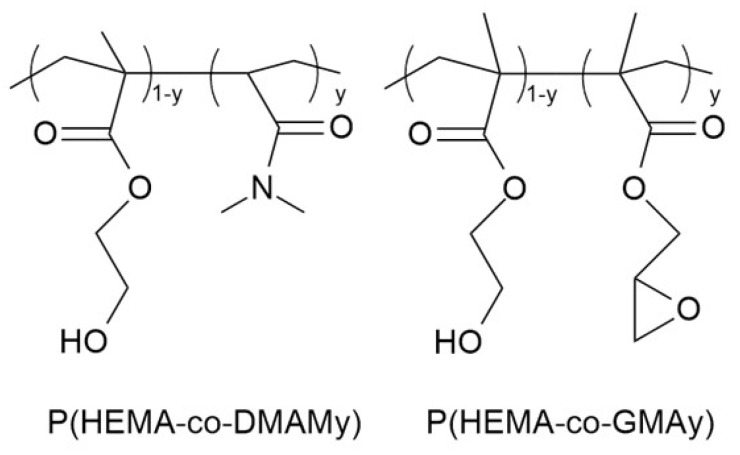
Chemical structures of P(HEMA-co-DMAMy) and P(HEMA-co-GMAy).

All HEMA-based copolymers were synthesized through free radical polymerization using azobisisobutyronitrile (AIBN) as the initiator, according to the experimental procedures described in the ‘Materials and Methods’ section. The structure of all copolymers is presented in Figure 1. The copolymers are denoted as P(HEMA-co-DMAMy) and P(HEMA-co-GMAy), where y are the molar percentages of DMAM or GMA units, respectively. All polymers were characterized by Proton Nuclear Magnetic Resonance (^1^H NMR) and Attenuated Total Reflection Fourier Transform Infrared Spectroscopy (ATR-FTIR).

**Figure 2:**
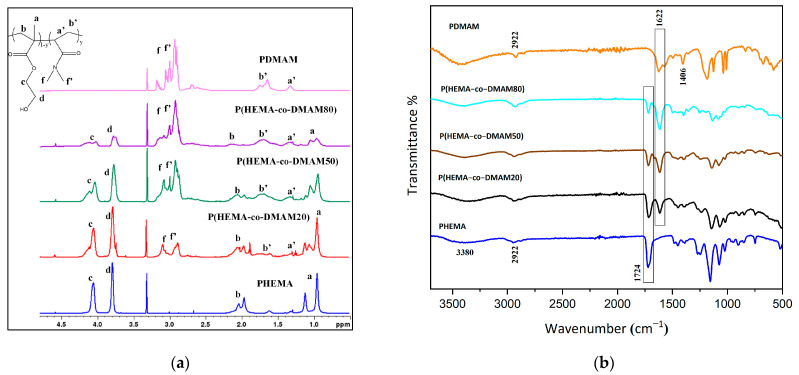
(**a**) ^1^H NMR spectra of the P(HEMA-co-DMAMy) copolymers in *d*_4_-MeOH. The spectra of the homopolymers PHEMA and PDMAM, are also presented.; (**b**) ATR-FTIR spectra of the P(HEMA-co-DMAMy) copolymers. The spectra of the homopolymers PHEMA and PDMAM, are also presented.

^1^H NMR was employed for the characterization of the P(HEMA-co-DMAMy) copolymers (Figure 2a). The ^1^H NMR study of the P(HEMA-co-DMAMy) copolymers and the homopolymers PHEMA and PDMAM was performed in *d*_4_-MeOH. The peaks within the range 0.80–1.20 ppm are attributed to the methyl protons (a) of HEMA. The protons of the backbone (-CH_2_-, b, b′; -CH, a′) of HEMA and DMAM are identified in the range 1.20–2.20 ppm. The peaks observed at ~3.70 and ~4.20 ppm are attributed to the methylene protons d and c of HEMA, respectively. The presence of DMAM moieties in the final copolymers was confirmed by the existence of the signals in the range ~2.80–3.20 ppm, which correspond to the methyl protons (f, f’) connected with the nitrogen atom in the DMAM units. The integrals of the signals at 3.70 ppm (d protons of HEMA units) and at 2.80–3.20 ppm (f,f′ protons of DMAM units) were used to calculate the molar ratio of HEMA and DMAM in the P(HEMA-co-DMAMy) copolymers. The results are summarized in Table 1. The calculated compositions of the copolymers are in good agreement with the feed composition of the reaction mixtures.

The ATR-FTIR spectra of the P(HEMA-co-DMAMy) copolymers, along with the spectra of the corresponding homopolymers, PHEMA and PDMAM, are presented in Figure 2b. A broad peak at ~3400 cm^−1^ corresponds to the stretching vibration band of the hydroxyl group (-OH) in HEMA. The band at 2922 cm^−1^ is attributed to the aliphatic C-H stretching vibrations of both HEMA and DMAM. The characteristic peak at 1724 cm^−1^, corresponding to the stretching vibrations of the carbonyl C=O group of HEMA is present in the spectrum of PHEMA homopolymer and in the spectra of all P(HEMA-co-DMAMy) copolymers, which affirms the presence of HEMA in the final copolymers. Moreover, the characteristic peak at 1622 cm^−1^ due to the vibration of the C=O groups of DMAM, is present in both PDMAM homopolymer and P(HEMA-co-DMAMy) copolymers, confirming the successful copolymerization of HEMA with DMAM. The relative variation of the intensities of these bands, 1724 and 1622 cm^−1^, respectively, is correlated with the concentration of each co-monomer.

**Figure 3:**
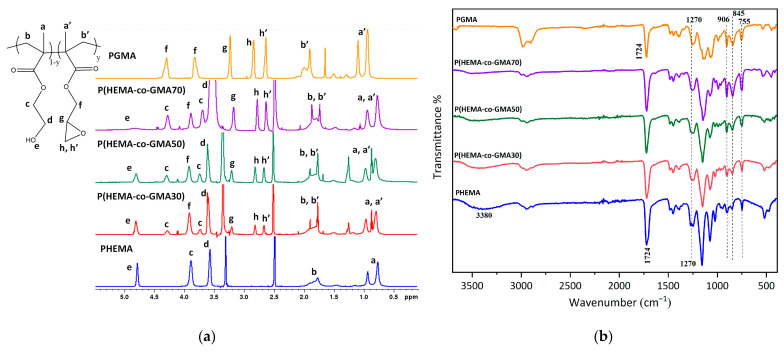
(**a**) ^1^H NMR spectra of the P(HEMA-co-GMAy) copolymers in *d*_6_-DMSO. The spectra of the homopolymers PHEMA (in *d*_6_-DMSO) and PGMA (in CDCl_3_), are also presented.; (**b**) ATR-FTIR spectra of the P(HEMA-co-GMAy) copolymers. The spectra of the homopolymers PHEMA and PGMA, are also presented.

The P(HEMA-co-GMAy) copolymers were characterized by ^1^H NMR and ATR-FTIR spectroscopy (Figure 3). In Figure 3a, the ^1^H NMR spectra of the P(HEMA-co-GMAy) copolymers are presented, accompanied by the spectra of the homopolymers PHEMA and PGMA. The successful copolymerization of HEMA with GMA was confirmed by the presence of characteristic peaks of GMA at 2.7 and 2.8 ppm, corresponding to the methylene protons (h, h′), and at 3.20 ppm, corresponding to the methine proton (g). The methyl protons (a, a′) of both HEMA and GMA are observed at 0.79 and 0.96 ppm. The protons of the backbone (-CH_2_-, b, b′) of HEMA and GMA can be identified at 1.70–2.00 ppm. The signal at a chemical shift of 4.75 ppm is attributed to the one proton of the hydroxyl group in the HEMA side chain (e). The peak at 3.60 ppm corresponds to the methylene protons (d) of HEMA, while the signals at a shift of 3.70–4.30 ppm are attributed to the methylene protons of HEMA (c) and GMA (f). The molar ratio of HEMA and GMA in the P(HEMA-co-GMAy) copolymers was calculated from the integrals of the signals at 0.96 and 0.79 ppm (a, a′ protons of HEMA and GMA), and at 3.20 ppm (g proton of GMA unit). As seen in Table 1, the composition of the copolymers is in a rather good agreement with the feed composition.

Figure 3b shows the ATR-FTIR spectra of the P(HEMA-co-GMAy) copolymers and the PHEMA and PGMA homopolymers. In all homopolymers and copolymers, the characteristic ester stretching vibration band of the carbonyl group (C=O) of HEMA and GMA units, is observed at 1724 cm^−1^. The characteristic absorption band of C-O-C, observed at ~1270 cm^−1^, and the aliphatic C-H stretching vibrations at the region 2830–3030 cm^−1^, are attributed to both HEMA and GMA. A broad peak at ~3400 cm^−1^, which corresponds to the stretching vibration band of the hydroxyl group (-OH) in HEMA, is present in all P(HEMA-co-GMAy) copolymers. Likewise, the peaks at 906, 845, and 755 cm^−1^, which are assigned to the vibrations of the epoxy ring of GMA, are also present in the copolymers, implying the successful copolymerization of HEMA with GMA. Additionally, it can be seen that the intensity of the peaks corresponding to GMA increase, while the peak of the hydroxyl group of HEMA (3400 cm^−1^) decreases, as the GMA content increases in the copolymers, confirming qualitatively the variation of the composition of the copolymers.

### 2.2. Preparation of WPU/Polymer Composites

**Scheme 1:**
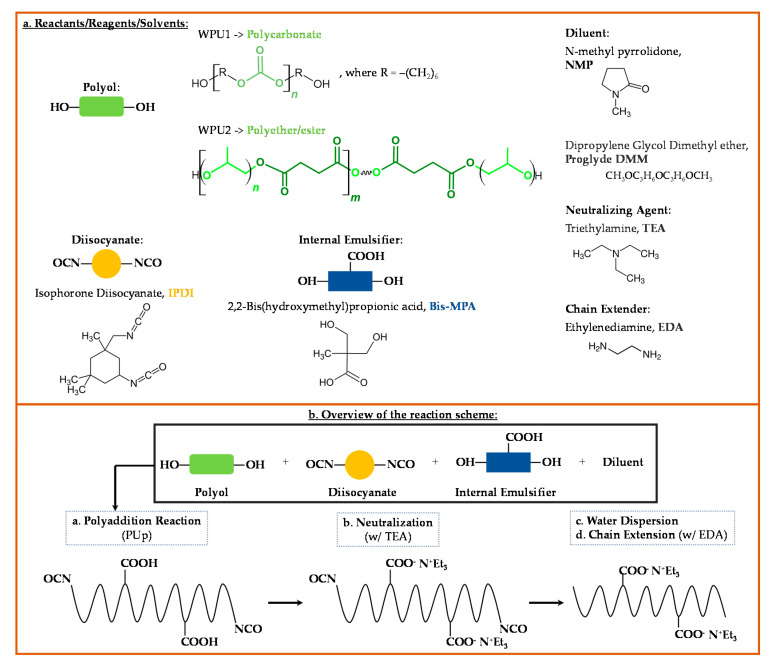
(**a**) Reagents, (**b**) Overview of the reaction scheme for the WPUs synthesis.

Two laboratory-made WPUs (namely WPU1 and WPU2) were used in this study for the preparation of WPU/polymer composite films. The main differences of the produced waterborne polyurethane dispersions WPU1 and WPU2 lie mostly in the type of the polyol used (polycarbonate polyol for WPU1 and polyether/ester polyol for WPU2), and the polyurethane resin being of pure polyurethane and hybrid polyurethane/alkyd nature for WPU1 and WPU2, respectively. For the development of the hybrid alkyd polyurethane dispersion WPU2, a certain amount of the polyether/ester polyol was replaced by two hydroxy modified alkyd resins, based on soybean oil and sunflower fatty acid. Following this methodology, the polymer chain of the produced resin contains randomly dispersed chains of alkyd resins, thus combining the properties of both structural components to enhance the final properties of the polyurethane dispersion. The overview of the reaction scheme for the WPUs synthesis is presented in Scheme 1, while the specifications of the formulated WPUs and selected physical properties are summarized in Table 2.

WPU dispersions were mixed with P(HEMA-co-DMAMy) or P(HEMA-co-GMAy) copolymers solutions, transferred on Teflon sheet, and left to dry at room temperature until full evaporation of solvents and film formation. The nomenclature used for the final products is WPUi/x polymer, where i = 1 or 2, and x is the mass percent of the copolymers in the WPU film (x = mass of polymer/mass of the solid content in PU of the WPU dispersions). Films with x = 10% *w*/*w* and 20% *w*/*w* were prepared. Both pure WPUs and WPU/polymer composites displayed homogeneous and smooth surfaces, as shown through Scanning Electron Microscopy (Figure S1). Moreover, preliminary Dynamic Mechanical Analysis studies showed that influence of the incorporation of the copolymers (at least at low contents) on the mechanical properties of the films is rather marginal (Figure S2).

**Figure 4:**
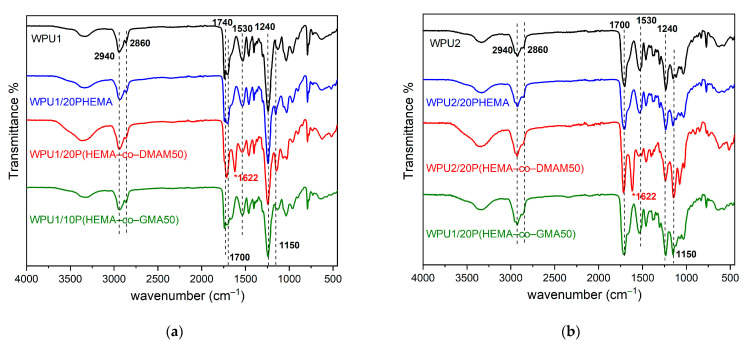
ATR-FTIR spectra of (**a**) WPU1 and (**b**) WPU2 films containing PHEMA, P(HEMA-co-DMAM50) and P(HEMA-co-GMA50) as additives.

The WPUs and WPU/polymer blend films were characterized by ATR-FTIR (Figure 4). In the spectra of both pure WPUs (Figure 4a,b), characteristic absorption peaks of polyurethanes can be detected (2940 cm^−1^: asymmetric stretching CH_2_ vibrations of alkanes; 2860 cm^−1^: symmetric stretching CH_2_ vibrations of alkanes; 1700 cm^−1^: C=O stretching; 1530 cm^−1^: bending vibration of N-H of urethane (Amide II); 1240 cm^−1^: C-O stretching; the peaks in the area 1100–1350 cm^−1^: stretching of O-C=O and C-N groups of urethanes). Moreover, it can be observed that in pure WPU1 film (Figure 4a), there is a peak at 1740 cm^−1^ that corresponds to non-hydrogen bonded C=O of urethane group. This peak is stronger compared to the peak at 1700 cm^−1^. In all WPU1/polymer composite films (Figure 4a), a new peak at 1150 cm^−1^ is detected, that is not present in the spectrum of pure WPU1. This peak is attributed to the stretching vibrations of C-O-C of HEMA moieties. As shown in Figure 4b, the same peak is present in the spectra of all films (including pure WPU2) and its signal tends to increase in the case composites, compared to pure WPU2. In both WPU1/P(HEMA-co-DMAM50) and WPU2/P(HEMA-co-DMAM50) blends (Figure 4a,b), the characteristic peak at 1622 cm^−1^ due to the vibration of the C=O groups of DMAM, is present, proving the successful incorporation of P(HEMA-co-DMAM50) in WPUs. Additionally, it seems that in the WPU1/PHEMA and WPU1/P(HEMA-co-GMA50) composite films (Figure 4a), the signal of the peak at 1700 cm^−1^ is enhanced, compared to the signal of the peak at 1740 cm^−1^, and this is attributed to the characteristic ester stretching vibration band of the carbonyl groups (C=O) of HEMA and GMA units in PHEMA and P(HEMA-co-GMA50) copolymer, at 1724 cm^−1^, which overlaps the polyurethane peaks in the specific area.

### 2.3. Study of Hydrophilicity

**Figure 5:**
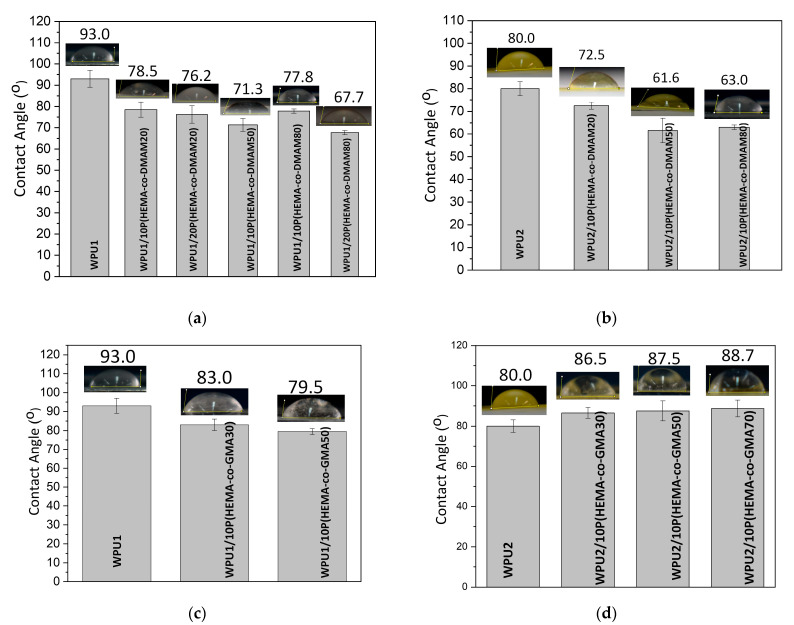
Water contact angles of WPU and WPU/polymer films: (**a**) WPU1/xP(HEMA-co-DMAMy); (**b**) WPU2/xP(HEMA-co-DMAMy); (**c**) WPU1/xP(HEMA-co-GMAy); (**d**) WPU2/xP(HEMA-co-GMAy).

As a consequence of the polar hydroxyl functionalities of HEMA units and amide functionalities of DMAM units, enabling hydrogen bonding with water molecules, the homopolymers and copolymers of these monomers are expected to be hygroscopic materials. This feature could offer water-responsiveness to the HEMA-based synthesized copolymers, and, consequently, affect the wettability of the as-prepared films and the envisaged water-mediated healing properties. Figure 5 shows the water contact angles of pure WPUS, WPU/xP(HEMA-co-DMAMy) as well as WPU/xP(HEMA-co-GMAy) films. Initially, it can be seen that among the pure WPU films, WPU1 displayed higher hydrophobicity compared to WPU2, with the water contact angle of WPU1 being 93.0° whereas the angle of WPU2 which was determined to be 80.0°, similar contact angle values reported for polyurethanes in the literature [25,61]. As shown in Figure 5a,b, the water contact angle decreases substantially with the addition of P(HEMA-co-DMAMy) copolymers, indicating the higher surface hydrophilicity of the WPU/xP(HEMA-co-DMAMy) composites as compared to pure WPUs films. This finding indicates that the wettability of the films is affected by the incorporation of the copolymers in the WPUs. Obviously, the hydrophilic P(HEMA-co-DMAMy) copolymers can attract the water molecules resulting in adsorption and penetration of water in the composite films. When the films with the more hydrophobic P(HEMA-co-GMAy) copolymers is studied, marginal changes are observed. In fact, in the case of the WPU1/xP(HEMA-co-GMAy) films (Figure 5c), the addition of the P(HEMA-co-GMAy) copolymers in WPU1 led to films with enhanced hydrophilicity, not so profound though, comparing with the results found in Figure 5a where the more hydrophilic P(HEMA-co-DMAMy) copolymers were used. On the contrary, Figure 5d demonstrates that the presence of the P(HEMA-co-GMAy) copolymers in WPU2 slightly enhances the hydrophobicity of the films. Indeed, it is seen that the water contact angle of WPU2 increases from 80.0° to 88.7°, when the P(HEMA-co-GMA70) copolymer with the highest hydrophobic content (70% mol in GMA moieties) is added in WPU2.

**Figure 6:**
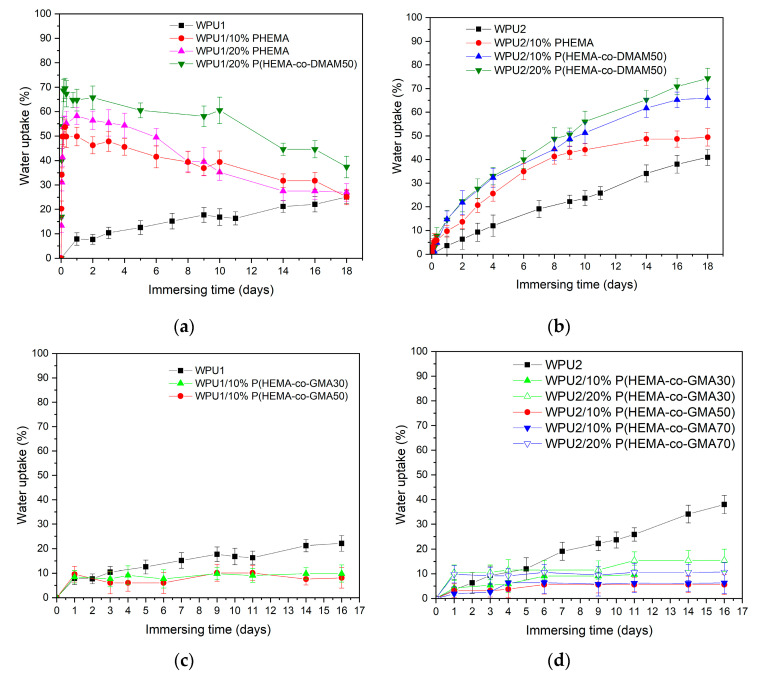

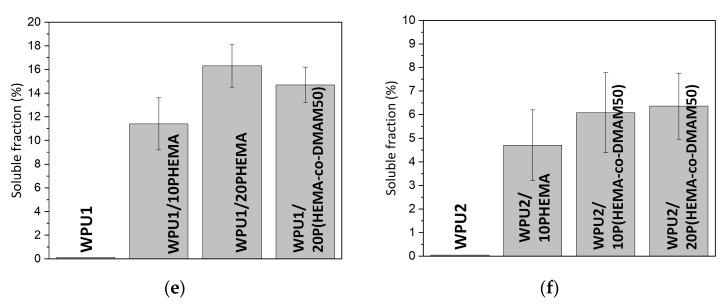
Water uptake of WPU and WPU/polymer films: (**a**) WPU1, WPU1/xPHEMA and WPU1/xP(HEMA-co-DMAMy); (**b**) WPU2, WPU2/xPHEMA and WPU2/xP(HEMA-co-DMAMy); (**c**) WPU1 and WPU1/xP(HEMA-co-GMAy); (**d**) WPU2 and WPU1/xP(HEMA-co-GMAy). Soluble fraction of (**e**) WPU1/xPHEMA and WPU1/xP(HEMA-co-DMAMy) films and, (**f**) WPU2/xPHEMA and WPU2/xP(HEMA-co-DMAMy) films.

The water sorption kinetics by the samples of WPU and WPU/HEMA-based copolymers are shown in Figure 6. Sorption of water by pure WPUs is the lowest among all studied films. The water uptake is about 25% and 40% for pure WPU1 and pure WPU2, respectively. This finding is probably correlated with the less hydrophobic character of the pure WPU2 film, as shown in the water contact angle results in Figure 5. When WPU1 is mixed with PHEMA homopolymer or P(HEMA-co-DMAMy) copolymers, the final composite films exhibit initially a high-water uptake, obtaining values up to 50%, 60%, and 85%, for the WPU1/10PHEMA, WPU1/20PHEMA, and WPU1/10P(HEMA-co-DMAM50) composites (Figure 6a), indicating the enhanced hydrophilicity of the films that makes them prone to water permeation, in qualitative agreement with the contact angle measurements observed for the WPU/xP(HEMA-co-DMAMy) films. However, after this initial swelling step, a tendency of all WPU1/xPHEMA and WPU1/xP(HEMA-co-DMAM50) films to gradually decrease water uptake values is observed, tending in some cases to meet finally the respective values of pure WPU1 films (see WPU1/10PHEMA and WPU1/20PHEMA films at 18 days). This decreasing tendency may be due to the dissolution of the water-soluble copolymers after long immersion time in water, affecting thus the weight and the hydrophilic behavior of the films. This hypothesis can be explained by the findings in Figure 6e, where the soluble fraction of the WPU1/xPHEMA and WPU1/xP(HEMA-co-DMAM50) films is quite high, taking values between 10% and 17%, while the soluble fraction of pure WPU1 film is zero as the film did not lose any of its initial weight after the completement of the water uptake study. In the case of the WPU2/xPHEMA and WPU2/xP(HEMA-co-DMAM50) films (Figure 6b), the behavior is different as the composite films display a continuous increase of the water uptake values, with the WPU2/xP(HEMA-co-DMAM50) films exhibiting the higher one. This indicates that the WPU2/xP(HEMA-co-DMAM50) films have higher wettability than the WPU2/xPHEMA composite films. Additionally, these films have a much lower soluble fraction (Figure 6f), compared to the findings of Figure 6e. Indeed, herein, the soluble fraction does not exceed the value of 6% for all studied samples, whereas the soluble fraction of pure WPU2 film is zero.

A totally different behavior is observed by the WPU/xP(HEMA-co-GMAy) composites. In both WPU1/xP(HEMA-co-GMAy) (Figure 6c) and WPU2/xP(HEMA-co-GMAy) (Figure 6d) cases, all composite WPU/polymer films display a water uptake lower than 15%, indicating the lower hydrophilicity of the films, in accordance with the higher water contact angle results observed, as compared to the corresponding WPU/xP(HEMA-co-DMAM50) films.

### 2.4. Self-Healing Property

**Figure 7:**
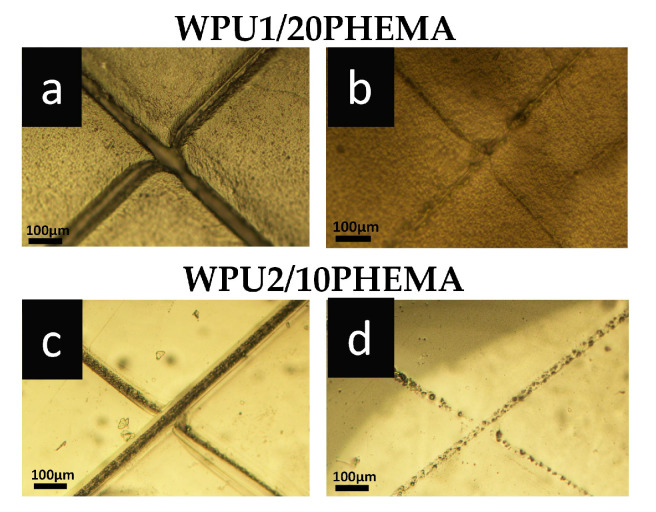
Optical microscopy photographs of (**a**,**b**) WPU1/20PHEMA (**c**,**d**) WPU2/10PHEMA films: images under damage (**a**,**c**) and healed (**b**,**d**) samples. Scale bars indicate 100 μm.

Figure 7 shows the self-healing process of WPU1/20PHEMA and WPU2/20PHEMA films, after the addition of droplets of water on the cut scratches. The optical microscope images of the healed samples were taken after full evaporation of the added water. As shown in Figure 7a,b, the scratch on the WPU1/20PHEMA film was healed at a large extend, whereas the scratch on the WPU2/20PHEMA film (Figure 7c,d) did not heal completely after addition of water on it.

**Figure 8:**
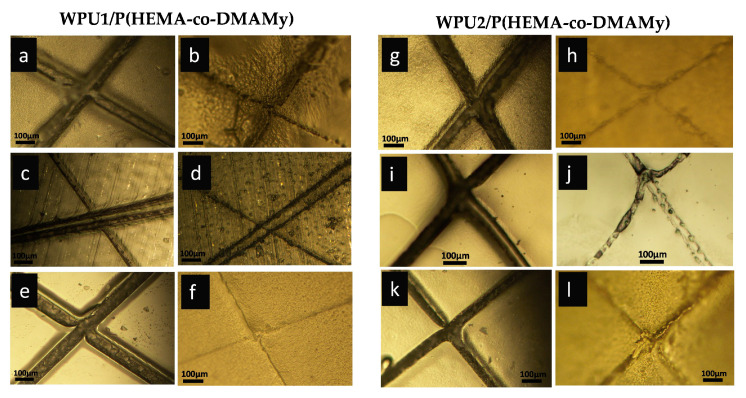
Optical microscopy photographs of (**a**,**b**) WPU1/20P(HEMA-co-DMAM20); (**c**,**d**) WPU1/20P(HEMA-co-DMAM50); (**e**,**f**) WPU1/20P(HEMA-co-DMAM80) and (**g**,**h**) WPU2/10P(HEMA-co-DMAM20); (**i**,**j**) WPU2/10P(HEMA-co-DMAM50); (**k**,**l**) WPU2/10P(HEMA-co-DMAM80) specimens: images under damage (**a**,**c**,**e**,**g**,**i**,**k**) and healed (**b**,**d**,**f**,**h**,**j**,**l**) samples. Scale bars indicate 100 μm.

It is expected that the hydrophilic character of the P(HEMA-co-DMAMy) copolymers will improve the water-mediated healing of the final WPU/xP(HEMA-co-DMAMy) composite films. Indeed, all WPU/xP(HEMA-co-DMAMy) films display improved healing efficiency after only once addition of water droplets on the cut (Figure 8). Moreover, in some cases (WPU1/20P(HEMA-co-DMAM80, Figure 8e,f, and WPU2/10P(HEMA-co-DMAM80), Figure 8k,l), the cross scratch was healed at a large extend or became almost invisible. As proved in Figure 6, the WPU/P(HEMA-co-DMAMy) films showed long term water absorption. Thus, it can be concluded that the systems under study are governed by a diffusion process which initiates from the surface of the films. Upon exposure to water, the damaged films can adsorb a quantity of water molecules. The water adsorption on the surface of the films leads to polymer chains’ plasticization that affects the chain dynamics [62]. The cut surfaces of the films heal due to the water-mediated polymer chain mobility, and now the diffusion of the polymer chains across the damaged region becomes probable and achievable. Furthermore, after the migration of the mobile polymer chains in the damaged interface, hydrogen-bonding interactions owed to both hydrogen-bond donors HEMA and DMAM components are enabled, leading to enhanced healing-efficiency. Therefore, the healability of these films is a combination of both diffusion and formation of hydrogen bonding.

**Figure 9:**
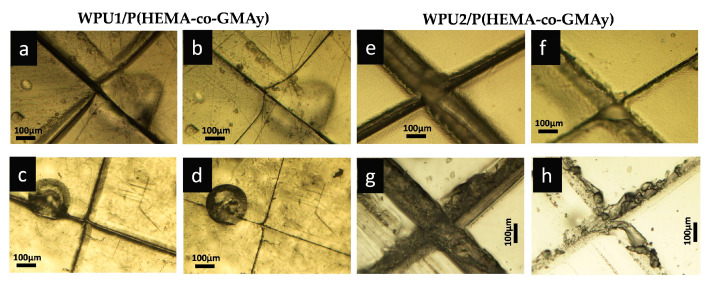
Optical microscopy photographs of (**a**,**b**) WPU1/10P(HEMA-co-GMA30); (**c**,**d**) WPU1/10P(HEMA-co-GMA50); (**e**,**f**) WPU2/10P(HEMA-co-GMA30); (**g**,**h**) WPU2/10P(HEMA-co-GMA50) specimens: images under damage (**a**,**c**,**e**,**g**) and healed (**b**,**d**,**f**,**h**) samples. Scale bars indicate 100 μm.

At a next step, the water-mediated self-healing performance of the WPU/PHEMA-co-GMAy) films was explored. As seen in Figure 9a–d, where the optical microscopy images of the WPU1/P(PHEMA-co-GMAy) films are shown, the cross scratch in the surface of the films was almost completely healed after the addition of small quantity of water on the cut area. It seems that self-healing is efficient for both copolymers used as additives in WPU1 (P(HEMA-co-GMA30) in Figure 9a,b; P(HEMA-co-GMA50) in Figure 9c,d). The improved hydrophilicity these films, supported by the contact angles measured in Figure 4c, seems to be the answer to the healing-behavior of this system. This hydrophilicity of the final composite films possibly allows the water molecules to provide a polymer chain mobility at a satisfying degree, enabling the polymer chains diffusion to the damaged area. After the cracked surfaces have come closer, possible mechanisms that can take place are hydrogen bonding due to HEMA moieties, hydrolysis of the epoxide groups of GMA by water, and/or reaction of the epoxide groups of GMA with groups like -NH_2_, -OH, or -COOH, that could be present in the polyurethane matrix.

The self-healing performance of WPU2/P(HEMA-co-GMAy) films is not as efficient as in the cases of the respective films based on WPU1. In Figure 9e–h, it is shown that the addition of droplets of water on the cut area did not provide water-assisted self-healing in a satisfying level. On the contrary, one can see that the cross cuts are just marginally healed. This observation can be corroborated by the hydrophobic character of the WPU2/P(HEMA-co-GMAy) films, which in Figure 5d exhibited higher water contact angles (>86.5°), compared to other composite films (WPU/P(HEMA-co-DMAMy), or WPU1/P(HEMA-co-GMAy) films), in combination with their very low water uptake ratios shown in Figure 5d. Thus, the hydrophobicity of the present films is a restraining factor of water-assisted self-healing.

## 3. Materials and Methods

### 3.1. Materials

The monomers HEMA (97%, Acros Organics, Illkirch, France), DMAM (>99%, TCI Chemicals, Zwijndrecht, Belgium), GMA (≥97%, Aldrich, Steinheim, Germany), and the initiator azobisisobutyronitrile (AIBN, Aldrich, Steinheim, Germany) were used as received. The solvents tetrahydrofuran (THF, anhydrous, p.a, ≥99.9%, Carlo Erba, Milano, Italy), *N*,*N*-dimethyl formamide (DMF, p.a, ≥99.8%, Macron fine chemicals, Center valley, PA, USA), hexane (p.a, ≥99.5%, Carlo Erba, Milano, Italy), ethanol (p.a, ≥99.5%, Carlo Erba, Milano, Italy), ethyl acetate (p.a, Macron fine chemicals, Center valley, PA, USA), and *N*-methyl pyrrolidone (NMP, Aldrich, Steinheim, Germany) were used as received without further purification. The deuterated solvents chloroform (CDCl3, 99.8% D), methanol (*d*_4_-MeOH, 99.9%) and dimethyl sulfoxide (DMSO-*d*_6_, 99.8%), were obtained from Eurisotop (Saint-Aubin, France). The solvents 1-butylpyrrolidin-2-one (Tamisolve, ≥99.5%) and di(propylene glycol) dimethyl ether (Proglyde DMM, ≥99.1%) were purchased from Aldrich (Steinheim, Germany). Ultrapure water was prepared by an Arium mini water purification system (Sartorius, Goettingen, Germany). The internal emulsifier 2,2-Bis(hydroxymethyl)propionic acid (Bis-MPA, 98%) was purchased from Perstorp Chemicals (Arnsberg, Germany). The neutralizing agent triethylamine (TEA, ≥99.5%), the chain extender ethylenediamine (EDA, ≥99.5%), isophorone diisocyanate (IPDI, 98%) and dibutyltin dilaurate (DBTL, 95%) were purchased from Aldrich (Steinheim, Germany).

### 3.2. Synthesis of Copolymers and WPUs

#### 3.2.1. Synthesis of P(HEMA-co-DMAMy) Copolymers

The synthesis of the copolymers was performed by free radical polymerization (FRP) of HEMA and DMAM monomers according to methodology reported in the literature [63]. For the synthesis of the copolymer P(HEMA-co-DMAM20), HEMA (5.00 mL, 41.10 mmol), DMAM (1.06 mL, 10.29 mmol), AIBN (1 mol% over the total monomer concentration, 84.0 mg, 5.1 × 10^−4^ mmol), and DMF (32.00 mL) were added into a round-bottom flask. The mixture was immersed in an oil bath at 65 °C and sealed under argon atmosphere. After 24 h, the reaction was stopped by exposing the mixture to air and lowering the temperature. The copolymer P(HEMA-co-DMAM20) was then purified by precipitation into an excess of ethyl acetate, filtered and dried in a vacuum oven at 40 °C overnight. Two more P(HEMA-co-DMAMy) copolymers were synthesized with different monomer proportions (y = 50, 80% moles DMAM).

#### 3.2.2. Synthesis of P(HEMA-co-GMAy) Copolymers

The P(HEMA-co-GMAy) copolymers were synthesized via FRP of HEMA and GMA monomers using AIBN as the initiator in THF at 70 °C [64]. A typical polymerization reaction, for example for the P(HEMA-co-GMA30), is the following: HEMA (10.00 mL, 82.20 mmol), GMA (4.67 mL, 35.15 mmol), AIBN (0.8 mol% over the total monomer concentration, 0.1543 g, 0.94 mmol), and THF (80.00 mL) were added in a round-bottom flask equipped with a magnetic stirrer and a reflux condenser. The mixture was degassed and left under argon atmosphere in an oil bath at 70 °C for 24 h. The final product was received by precipitation in hexane, filtered, washed with hexane, and dried in a vacuum oven at 40 °C for 24 h. Three P(HEMA-co-GMAy) copolymers were totally synthesized, with GMA feed composition y = 30%, 50%, and 70% moles, respectively.

#### 3.2.3. Synthesis of the Waterborne Polyurethane Dispersions WPU1 and WPU2

The waterborne polyurethane dispersions were developed through the prepolymer process, consisting of the following four steps: (a) formation of a water-dispersible, NCO-terminated PolyUrethane Prepolymer (PUp) from a polyol (MW: 1000 g/mol) and IPDI, in the presence of DBTL catalyst, with the aid of the solubilizing agent/internal emulsifier Bis-MPA, and a diluent for viscosity control, namely Proglyde DMM and NMP for WPU1 and WPU2, respectively; (b) neutralization of the PUp with TEA; (c) dispersion of the PUp in water at high shear rate (500–2000 rpm), depending on the PUp viscosity; and (d) chain extension of the neutralized, water-dispersible PUp with EDA.

### 3.3. Preparation of WPU/Polymer Composites

The P(HEMA-co-DMAMy) copolymers were dissolved in ultrapure water (for y = 50, 80% mol) or ethanol (for y = 20% mol). The PHEMA homopolymer was dissolved in ethanol. The P(HEMA-co-GMAy) copolymers were dissolved in a mixture 1:1 *w*/*w* of Tamisolve and Proglyde solvents. In all cases, the concentration of polymers was 10% *w*/*v*. The polymer solutions were homogeneously mixed with the waterborne polyurethane dispersions. The mixtures were poured on Teflon sheet and dried for 10 days at room temperature. The nomenclature used for the final products is WPUi/x polymer, where i = 1 or 2, and x is the mass percent of the copolymers in the WPU film (x = mass of polymer/mass of the solid content in PU of the WPU dispersions). Films with x = 10% *w*/*w* and 20% *w*/*w* were prepared. The dried composite films had a thickness of ~0.3 mm. Films of pure WPU dispersions were also prepared in a similar fashion for reasons of comparison.

### 3.4. Characterization Techniques

#### 3.4.1. Proton Nuclear Magnetic Resonance (^1^H NMR)

^1^H NMR study (at 400 MHz and at 25 °C) of all homopolymers and copolymers was accomplished using a Bruker AVANCE DPX 400 spectrometer (Billerica, MA, USA).

#### 3.4.2. Attenuated Total Reflection Fourier Transform Infrared Spectroscopy (ATR-FTIR)

The ATR-FTIR spectra of the homopolymers and the HEMA-based copolymers were recorded on a Bruker Platinum spectrometer (Billerica, MA, USA).

#### 3.4.3. Water Contact Angle

The hydrophilic character of WPU/polymer composite films was investigated at room temperature by water contact angle measurements. Specifically, a 20 μL droplet of ultrapure water was pipetted onto the surfaces of the pure WPU or the WPU/polymer films and the contact angles were measured using ImageJ software. 

#### 3.4.4. Water Uptake and Soluble fraction

Water uptake of the WPU/polymer composite films was determined by immersing the films in ultrapure water at room temperature. At regular time intervals, water was gently wiped off the surface of the films using filter paper, and the films were weighted. The water uptake ratio was calculated by the following Equation (1):

Water uptake (%) = [(W − W_d_)/W_d_] × 100 (1)

where W_s_ is the weight of the swollen films at different immersing times, and W_d_ is the initial weight of the dried films.

After the water uptake study was over, the films were dried under vacuum at 50 °C, then the soluble fraction of the films was calculated using Equation (2):

Soluble fraction (%) = [(W_f_ − W_d_)/W_d_] × 100 (2)

where W_f_ is the weight of the dried films after the water uptake study, and W_d_ is the weight of the dried initial films before the water uptake study.

#### 3.4.5. Self-Healing Tests

To study the self-healing behavior, the polyurethane/polymer films were scratched with a razor blade. Afterwards, droplets of water were added on the scratch using a micropipette and left to dry at room temperature. The healing process of the films was monitored by a Nikon Eclipse L150 optical microscope (and Nikon’s NIS-Elements DS-U3 software) (Nikon metrology, Paris, France).

#### 3.4.6. Viscosity Measurements

The viscosity measurements of the WPUs were performed using a Brookfield RVDV-II+Pro viscometer, at room temperature and a shear rate of 100 rpm.

#### 3.4.7. Minimum Film Forming Temperature (MFFT)

The minimum film-forming temperature is the minimum temperature at which a waterborne synthetic latex or emulsion will coalesce when laid on a substrate as a thin film. When this process occurs, a clear transparent film is formed. At temperatures below the MFFT, drying will result in a white, powdery, cracked film. The MFFT measurements were performed with a Rhopoint instruments’ MFFT.

## 4. Conclusions

In summary, two series of HEMA-based copolymers were synthesized by free radical copolymerization and investigated as potential self-healing agents of waterborne polyurethane resins (WPU). The copolymers of HEMA with the hydrophilic DMAM, P(HEMA-co-DMAMy), combine characteristics like hydrogen-bonding ability of both structural units and enhanced hydrophilicity, while the copolymers of HEMA with the hydrophobic, epoxy-bearing GMA, P(HEMA-co-GMAy), are hydrophobic copolymers with dual functionality: hydrogen-bonding owing to HEMA units and reactivity through the nucleophilic ring opening reactions of the pendent epoxide group of GMA units. WPU/polymer composite films were readily prepared by mixing two different WPU dispersions (WPU1 and WPU2) with polymer solutions, followed by solution casting. The addition of P(HEMA-co-DMAMy) copolymers in both WPUs, led to films with enhanced surface hydrophilicity and water-uptake efficiency, as seen by water contact angle and water sorption kinetics measurements. On the other hand, the use of P(HEMA-co-GMAy) as additives resulted in less hydrophilic films with a lower water uptake. The self-healing efficacy of the WPU/polymer composites was explored upon addition of a small quantity of water as external healing trigger of the damaged area. Self-healing was mainly governed by mechanisms like polymer chain diffusion process and hydrogen-bonding, and epoxy-ring opening reactions originating from GMA. Depending on the WPU used, both copolymer series exhibited self-healing capabilities, which were more pronounced in the case of the hydrophilic P(HEMA-co-DMAMy) copolymers. The room-temperature, water-assisted self-healing ability of the developed composites is a green procedure and makes these materials attractive candidates for various indoor every-day practical applications, having the advantage of performance at room temperature and atmosphere, without using severe conditions for repair. For example, self-healing of WPU/polymer composites coatings could be accomplished by simply spraying water on the coating surface.

## Figures and Tables

**Table 1:** Characterization results for the copolymers.

P(HEMA-co-DMAMy)		
Polymers	Feed Composition (% mol DMAM)	^1^H NMR Composition (% mol GMA)
P(HEMA-co-DMAM20)	20.0	24.0
P(HEMA-co- DMAM 50)	50.0	51.2
P(HEMA-co- DMAM 80)	80.0	73.6
**P(HEMA-co-GMAy)**		
**Polymers**	**Feed Composition** **(% mol GMA)**	**1H NMR Composition** **(% mol GMA)**
P(HEMA-co-GMA30)	30.0	33.0
P(HEMA-co-GMA50)	50.0	45.5
P(HEMA-co-GMA70)	70.0	76.5

**Table 2:** Specifications/Physical Properties of the formulated/developed WPUs.

WPU Code	WPU Type	Polyol Type	NCO/OH	Solvent	NV ^a^ %	pH	AN ^b^ (mgKOH/g)	MFFT (°C)	Viscosity (cP)	Storage Stability (Months)
WPU1	Pure	polycarbonate	1.4	Proglyde DMM	37.30	7.20	27.30	−3.6	250.8	>12
WPU2	Hybrid Alkyd	polyether-ester	1.33	NMP	35.70	7.35	25.10	−6.0	42.4	>12

^a^ Non-volatile content; ^b^ Acid number.

## Data Availability

The data presented in this study are available on request from the corresponding author.

## Supplementary Materials

The following supporting information can be downloaded at: https://www.mdpi.com/article/10.3390/ijms24032575/s1, Figure S1: SEM images of (a) pure WPU1, (b) pure WPU2, (c) composite WPU1/20P(HEMA-co-DMAM50), and (d) composite WPU2/20P(HEMA-co-DMAM50) polyurethane film surfaces.; Figure S2: Storage modulus (E’, closed symbols) and loss modulus (E’’, open symbols) for pure WPU1 film, WPU1/10PHEMA composite film and WPU1/10PHEMA composite film.
