# Supplementary material for: Room-Temperature Self-Healable Blends of Waterborne Polyurethanes with 2-Hydroxyethyl Methacrylate-Based Polymers
Source: Int J Mol Sci. 2023 Jan 29;24(3):2575. doi: 10.3390/ijms24032575 (PMC9916575; doi:10.3390/ijms24032575)
Supplement: Supplementary file 1 [file ijms-24-02575-s001.zip › ijms-2160481-supplementary.pdf]

Supporting Information

Room-temperature self-healable blends of waterborne polyurethanes with 2-hydroxyethyl methacrylate-based polymers

Ioanna Tzoumani [1], Zacharoula Iatridi [1], Athena M. Fidelli [2], Poppy Krassa [2], Joannis K. Kallitsis [1], Georgios Bokias [1,*]

1. Department of Chemistry, University of Patras, GR-26504, Patras, Greece;
2. Megara Resins Anastassios Fanis S.A., Vathi Avlidas, GR-34100 Evia, Greece

* Correspondence: bokias@upatras.gr

The morphology of the obtained films was investigated by scanning electron microscopy (SEM) using a Zeiss ZUPRA 35 VP–FEG instrument (Carl Zeiss, Germany), operating at 5–20 keV. The films were coated with a conductive Au film through sputtering.

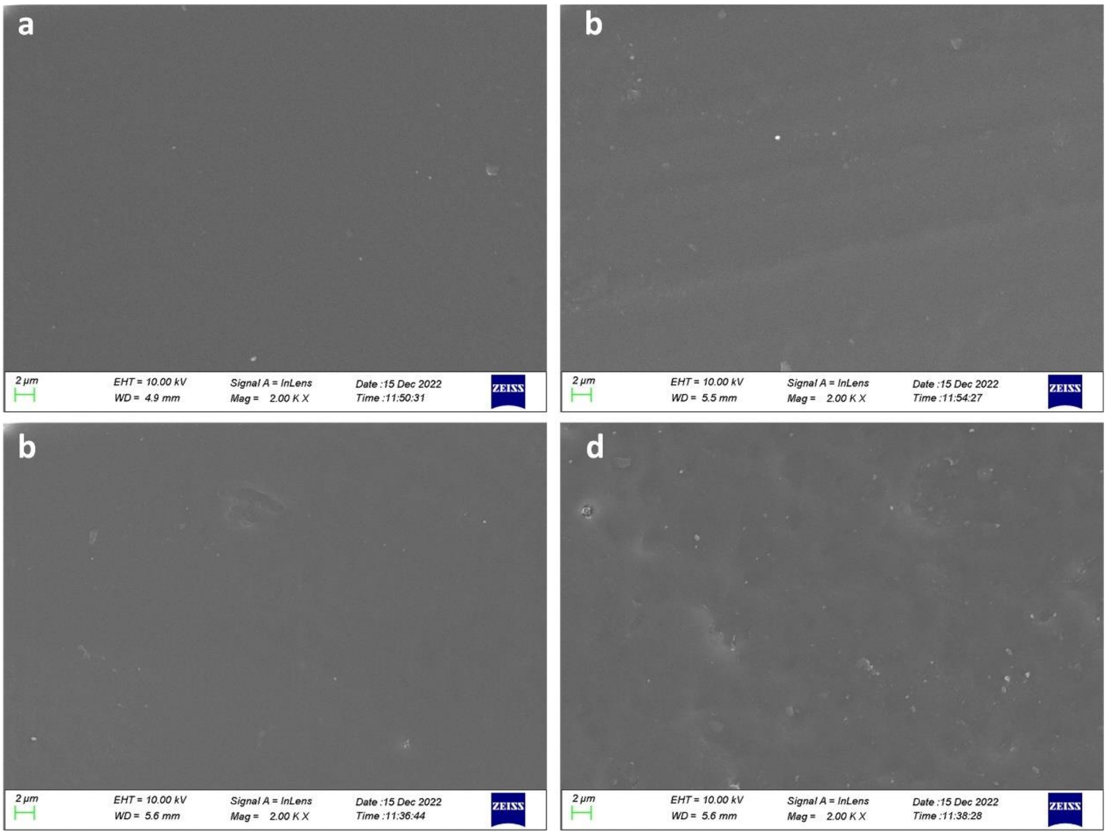

Figure S1. SEM images of a) pure WPU1, b) pure WPU2, c) composite WPU1/20P(HEMA–co–DMAM50) and d) composite WPU2/20P(HEMA–co–DMAM50) polyurethane film surfaces.

Dynamic mechanical analysis (DMA) was performed with a solid–state analyzer RSA II, Rheometric Scientific L.t.d. (West Yorkshire, UK) at 10Hz, in the temperature range of –20 °C up to 200 °C with a heating rate of 3 °C/min.

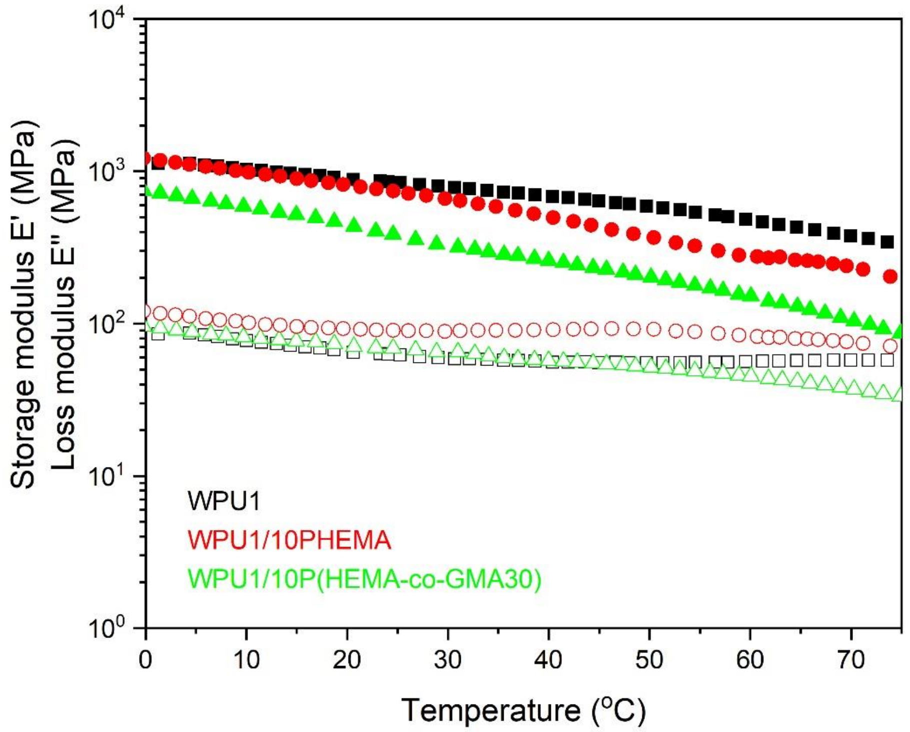

Figure S2. Storage modulus (E′, closed symbols) and loss modulus (E′′, open symbols) for pure WPU1 film (black, ■), WPU1/10PHEMA composite film (red, ●) and WPU1/10PHEMA composite film (green, ▲).
